# Transcript isoform switching during embryonic and post-hatch development of broiler breast muscle

**DOI:** 10.1016/j.psj.2026.107583

**Published:** 2026-08-05

**Authors:** Nabeel Alnahhas

**Affiliations:** a Department of Animal Science, Faculty of Agricultural and Food Sciences, Université Laval, 2425 Rue de l’Agriculture, Quebec City, G1V 0A6, Quebec, Canada; b Swine and Poultry Infectious Diseases Research Centre, Faculty of Veterinary Medicine, Université de Montréal, 3200 Rue Sicotte, St-Hyacinthe, J2S 2M2, Quebec, Canada

**Keywords:** Alternative splicing, Isoform switching, RNA-seq, Differential transcript usage, Broiler muscle development

## Abstract

Gene-level analysis of RNA-Seq data provides only a partial view of muscle transcriptional regulation because gene isoforms may be differentially used without changes in total gene expression. This study evaluated the extent and potential role of differential transcript usage (**DTU**) during breast muscle development from the late embryonic stage to market age. Publicly available RNA-Seq data from breast muscle of Cornish, White Plymouth Rock, and their crossbred progeny sampled at embryonic day 17 (**ED17**) and post-hatch days 1, 21, and 42 were analyzed using The R package *IsoformSwitchAnalyzeR* (*n* = 6 samples per genetic background and stage, total = 72 samples). The highest number of isoform switches (|Δ isoform fraction| > 0.1 and FDR < 0.05) occurred during ED17-to-D1 transition (933 switches), followed by D1-to-D21 (631 switches), whereas only 46 occurred between D21 and D42. Exon skipping, alternative transcription start sites, and alternative transcription termination sites were the most frequent splicing events associated with these switches (*n* = 1,042, 964, and 817, respectively). Changes in coding sequence completeness, intrinsically disordered regions, and protein domains were the most frequently predicted functional consequences (*n* = 497, 472, and 461 events, respectively). Gene ontology enrichment (*P* < 0.05) indicated that ED17-to-D1 switching genes were involved in sarcomeric and cytoskeletal organization, redox and metabolic adaptation, signaling, chromatin regulation, and RNA processing, with top genes including OBSCN, DTNA, ABLIM3, and LIMCH1. D1-to-D21 switching genes were associated with cytoskeletal restructuring, adhesion and mechanosensing, RNA processing, protein turnover, autophagy, metabolic adaptation, and stress signaling, with TNNT3, FHL1, and FHL3 among the top-ranked genes. Although fewer genes exhibited isoform switching during the D21-to-D42 transition, the top-ranked genes included MYH1B, SMYD1, and MRTFA, which are involved in contractile or muscle regulatory processes. In conclusion, isoform switching represents an additional layer of transcriptional regulation of muscle development and it may contribute to hatch-related maturation, rapid post-hatch growth, and later refinement of contractile function. Future studies should experimentally validate candidate switches and their predicted functional consequences.

## Introduction

Poultry meat is expected to account for 52% of worldwide meat consumption by 2030 (Sammari et al., 2023). To meet this increasing demand, the poultry industry has relied on genetic selection, improved nutritional strategies, and enhanced flock management to increase production efficiency. Breast meat yield (**BMY**) is one of the most economically important traits in the poultry industry, particularly in North American and European markets, where consumers favor meat products with high protein and low fat contents (Kuttappan et al., 2016). Consequently, genetic selection programs have placed strong emphasis on improving growth performance, carcass yield, and breast muscle development. In their study, Zuidhof et al. (2014) reported that, from 1957 to 2005, the yield of the *Pectoralis major* muscle increased by 79% and 85% in male and female broilers, respectively, while the yield of the *Pectoralis minor* muscle increased by 30% and 37%, respectively, as a result of continued selection.

Muscle growth during embryogenesis occurs primarily through hyperplasia, corresponding to an increase in the number of myofibers. At hatch, the number of myofibers is largely fixed, and post-hatch muscle growth occurs mainly through hypertrophy, characterized by increases in myofiber length and diameter (Velleman, 2015; Velleman, 2023). However, excessive hypertrophic growth has been associated with reduced capillary density, hypoxia, reduced glycolytic reserves, perturbations in muscle energy metabolism, and oxidative stress, all of which contribute to the development of breast muscle myopathies in broiler chickens (Kuttappan et al., 2016; Petracci et al., 2019; Alnahhas et al., 2023). Although research over the last decade has substantially improved our understanding of the mechanisms underlying muscle growth, meat quality, and growth-related myopathies (Velleman, 2019; Velleman, 2023), the triggering factors leading to meat quality defects and breast muscle abnormalities remain incompletely understood. This highlights the need for further investigation of the genetic and molecular regulation of normal muscle growth.

In recent years, transcriptomic studies have been increasingly used to unravel the transcriptional regulation of muscle growth, development, and myopathies in broiler chickens under diverse experimental and production conditions (Papah et al., 2018; Brothers et al., 2019; Pampouille et al., 2019; Malila et al., 2020; Achour et al., 2025). While these studies have considerably advanced our understanding of muscle biology and muscle abnormalities, they have primarily examined transcriptional regulation at the gene level using differential gene expression (**DGE**) analysis. However, in higher eukaryotes, multi-exon genes can produce multiple transcript isoforms through alternative splicing and/or the use of alternative transcription start or termination sites (Froussios et al., 2019). Because transcript isoforms can differ in coding potential, regulatory features, stability, localization, and functional output, differential transcript usage (**DTU**) analysis provides a complementary layer of resolution to DGE by detecting shifts in isoform composition within genes. Thus, DTU analysis can reveal transcript-level regulatory mechanisms that may contribute to cell growth and maturation but remain hidden in gene-level analyses (Cmero et al., 2019).

Therefore, the objective of the present study was to characterize changes in transcript usage during broiler breast muscle development, assess the predicted functional consequences of isoform switching at the protein level, and identify transcript-level regulatory mechanisms underlying muscle growth that are not captured by conventional gene-level RNA-Seq analysis.

## Materials and methods

### Ethics statement

This study was based exclusively on a publicly available dataset and did not involve the collection of new animal samples. Therefore, no additional ethical approval was required.

### Dataset description

This study used publicly available RNA-Seq data generated by Huang et al. (2025) from a temporal atlas of broiler breast muscle development. Briefly, the dataset consisted of bulk RNA-seq libraries generated from breast muscle samples collected from three broiler genetic groups: Cornish purebred chickens (**CC**), White Plymouth Rock purebred chickens (**RR**), and their crossbred progeny (**CR**). Samples were collected on embryonic day 17 (**ED17**) and post-hatching days 1, 21, and 42, representing late embryonic development, hatch, juvenile growth, and market age, respectively. The dataset included 72 samples in total, with six biological replicates per genetic group at each developmental stage. Libraries were sequenced on an Illumina NovaSeq 6000 platform, generating paired-end 150-bp reads. Raw sequencing data were retrieved from the NCBI Sequence Read Archive under accession number SRP560363. The original study included an in-depth technical validation of the dataset, reporting an average Q30 value of 94.16% across the 72 RNA-seq libraries and 20.06 to 28.69 million raw reads per sample, with a mean of 21.22 million reads. These sequencing metrics support the reliability and suitability of the dataset for downstream transcriptomic analyses (Huang et al., 2025). The original study also provided summary statistics of body weight (**BW**) and breast muscle weight (**BMW**) of birds from the three genetic backgrounds at each sampling timepoint.

### Bioinformatics

***Read trimming and transcript-level quantification***. Raw sequencing reads were processed with *fastp* (Chen et al., 2018) using default parameters to assess read quality, remove low-quality reads, and trim adapter sequences. Transcript-level quantification was then performed with the *Salmon* pseudo-aligner (Patro et al., 2017; https://combine-lab.github.io/salmon/) using default settings with the additional –seqBias option to account for sequence-specific biases introduced during library preparation and the –gcBias option to correct for fragment-level GC-content bias. Transcript quantification was performed against the Ensembl release 113 chicken transcriptome corresponding to the GRCg7b assembly, which was the most recent Ensembl annotation available when the transcriptome reference was selected and was used consistently throughout all quantification and downstream analyses.

***Importing counts and preprocessing***. Differential transcript usage and isoform-switching analyses were conducted in R version 4.5.1 using version 2.10.0 of the *IsoformSwitchAnalyzeR* framework (Vitting-Seerup and Sandelin, 2019). First, transcript-level abundance estimates obtained from *Salmon* were imported into R using the *importIsoformExpression* function, which also applies inter-library normalization using the scaled transcripts per million method (*scaledTPM*). Next, a *switchAnalyzeRlist* object was generated using the *importRdata* function by combining the transcript-level count matrix, abundance matrix, experimental design matrix, transcriptome FASTA file (Gallus_gallus.bGalGal1.mat.broiler.GRCg7b.cdna.all.fa.gz), and genome annotation file (Gallus_gallus.bGalGal1.mat.broiler.GRCg7b.113.gtf.gz). The design matrix included breed and developmental stage and was used to define the comparisons of interest. Isoform fractions (**IF**) were calculated as the relative contribution of each isoform to the total expression of its corresponding gene.

The annotation file was used to add transcript structure and coding-region information to the *switchAnalyzeRlist* object, including open reading frame (**ORF**) coordinates, coding sequence regions, transcript start and end positions, ORF length, the exons containing the start and stop codons, the distance from the stop codon to the last exon-exon junction, and the predicted presence of premature termination codons (**PTC**). This information was subsequently used for downstream isoform-switching and functional consequence analyses.

Next, a principal component analysis (**PCA**) was conducted on transcript-level normalized log2 counts per million (Log2CPM) using the *prcomp* function from base R. The *preFilter* function from *IsoformSwitchAnalyzeR* was then applied using default parameters to remove lowly expressed genes and isoforms unlikely to contribute to downstream isoform-switching analyses. Briefly, genes with expression levels below 1 TPM were removed, and isoforms were retained for downstream analysis when they showed a minimum differential IF (**dIF**) of 0.1 between conditions.

***Differential transcript usage analysis***. Isoform switch analysis was then conducted on the filtered data using the *isoformSwitchTestSatuRn* function. The *SatuRn* method uses a quasi-binomial generalized linear model framework to test for differential transcript usage by modelling the relative usage of each transcript compared with other transcripts from the same gene between conditions of interest (Gilis et al., 2021). To control the number of false-positive findings, *SatuRn* estimates *P*-values using an empirical null distribution, followed by multiple-testing correction using the Benjamini-Hochberg false discovery rate (**FDR**) method (Gilis et al., 2021). Significant isoform switches were defined as those with an FDR < 0.05 and an absolute dIF > 0.1 between conditions.

***Predicting consequences of DTU and alternative splicing (AS) patterns driving them***. The *extractSequence* function from *IsoformSwitchAnalyzeR* was used to extract the nucleotide sequences of significantly switching isoforms by retrieving and concatenating the reference genome sequences corresponding to their genomic coordinates. The same function was also used to translate the nucleotide sequence of annotated ORFs into amino acid sequences. Extracted nucleotide and amino acid sequences were exported as separate FASTA files and used for downstream prediction of alternative splicing patterns and functional consequences, respectively.

Functional consequences of isoform switching were predicted using external tools integrated into the *IsoformSwitchAnalyzeR* workflow. Coding potential was assessed using Coding *Potential Calculator 2.0* (Kang et al., 2017). Protein domains were predicted using the *Pfam* protein database (Bateman et al., 2004). Signal peptides were predicted using *SignalP* version 6.0 (Nielsen et al., 2024), while intrinsically disordered regions (IDRs) were predicted using *NetSurfP* version 3.0 (Høie et al., 2022). Protein topology, including transmembrane regions and orientation relative to the cell membrane, was predicted using *DeepTMHMM* version 1.0 (Hallgren et al., 2022). Subcellular localization was predicted using *DeepLoc* version 2.1 (Ødum et al., 2024).

The outputs from these external tools were imported into the *IsoformSwitchAnalyzeR* framework using the *isoformSwitchAnalysisPart2* function. In addition to integrating predicted functional consequences, this function was used to identify alternative splicing patterns associated with significant isoform switches.

The *extractSplicingSummary* and *extractConsequenceSummary* functions were used to summarize individual alternative splicing events and predicted functional consequences for each gene or switch. Enrichment of specific alternative splicing event types and predicted functional consequences was evaluated using the *extractSplicingEnrichment* and *extractConsequenceEnrichment* functions, respectively. Finally, comparisons of enrichment patterns between conditions were conducted using the *extractSplicingEnrichmentComparison* and *extractConsequenceEnrichmentComparison* functions.

***Differential gene expression (DGE) analysis.*** In parallel with the DTU analysis, differential gene expression (DGE) analysis was performed using the same RNA-Seq dataset with the *edgeR* package version 4.8.2 (Robinson et al., 2010). Transcript-level counts were imported into R, summed to obtain gene-level counts, normalized, and analyzed using the same developmental contrasts as those tested in the DTU analysis: D1 vs. ED17, D21 vs. D1, and D42 vs. D21, corresponding to the hatch, early growth, and late growth transitions, respectively. Genes with an absolute log2 fold change greater than 0.5, corresponding to approximately a 1.4-fold change, and an FDR < 0.05 were considered differentially expressed genes (**DEGs**). The DGE results were then incorporated into the *IsoformSwitchAnalyzeR* framework for downstream analyses.

***Gene ontology (GO) enrichment analysis.*** Genes showing significant isoform switching between developmental stages were subjected to GO enrichment analysis using the DAVID bioinformatics platform (Dennis et al., 2003; https://davidbioinformatics.nih.gov/). Enrichment was assessed separately for the three GO domains: biological process (**BP**), molecular function (**MF**), and cellular component (**CC**). Enriched terms (*P* < 0.05) were used to identify biological functions, molecular activities, and cellular localizations overrepresented among genes undergoing isoform switching.

***Analysis of top-ranked switching genes***. Genes were ranked within each developmental transition according to their isoform-switching significance. Among the top-ranked genes, those showing predicted functional consequences and known relevance to muscle development, structure, metabolism, or function were selected as representative examples. Switch plots were generated using the *switchPlot* function from *IsoformSwitchAnalyzeR* to visualize changes in isoform usage, transcript structure, and predicted functional features. The full bioinformatics pipeline is illustrated in Supplementary Fig. S1.

## Results

### Principal component analysis (PCA)

**Fig. 1:**
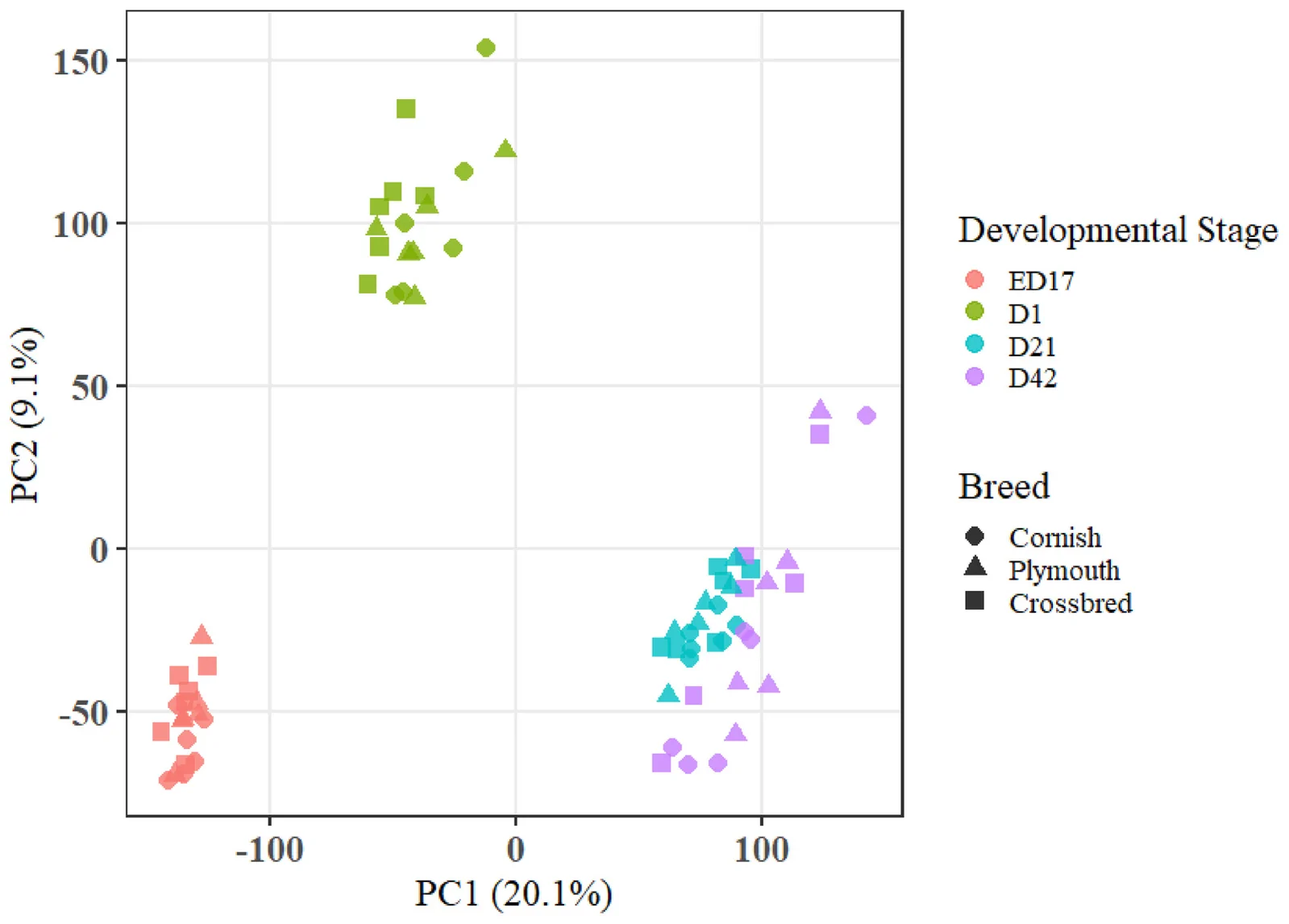
Principal component analysis of transcript-level expression data. Breast muscle samples were collected at embryonic day 17 (ED17) and post-hatch days 1, 21, and 42 from Cornish, White Plymouth Rock, and their crossbred progeny. Colors indicate developmental stage, and shapes indicate genetic group.

The PCA (Fig. 1) revealed a strong separation of samples according to developmental stage, indicating that age was the main source of transcriptomic variation in the dataset. In contrast, separation according to genetic background was comparatively modest and appeared mainly as within-stage substructure. PC1 and PC2 explained 20.1% and 9.1% of the total variance, respectively.

### Differential transcript usage (DTU) analysis

**Fig. 2:**
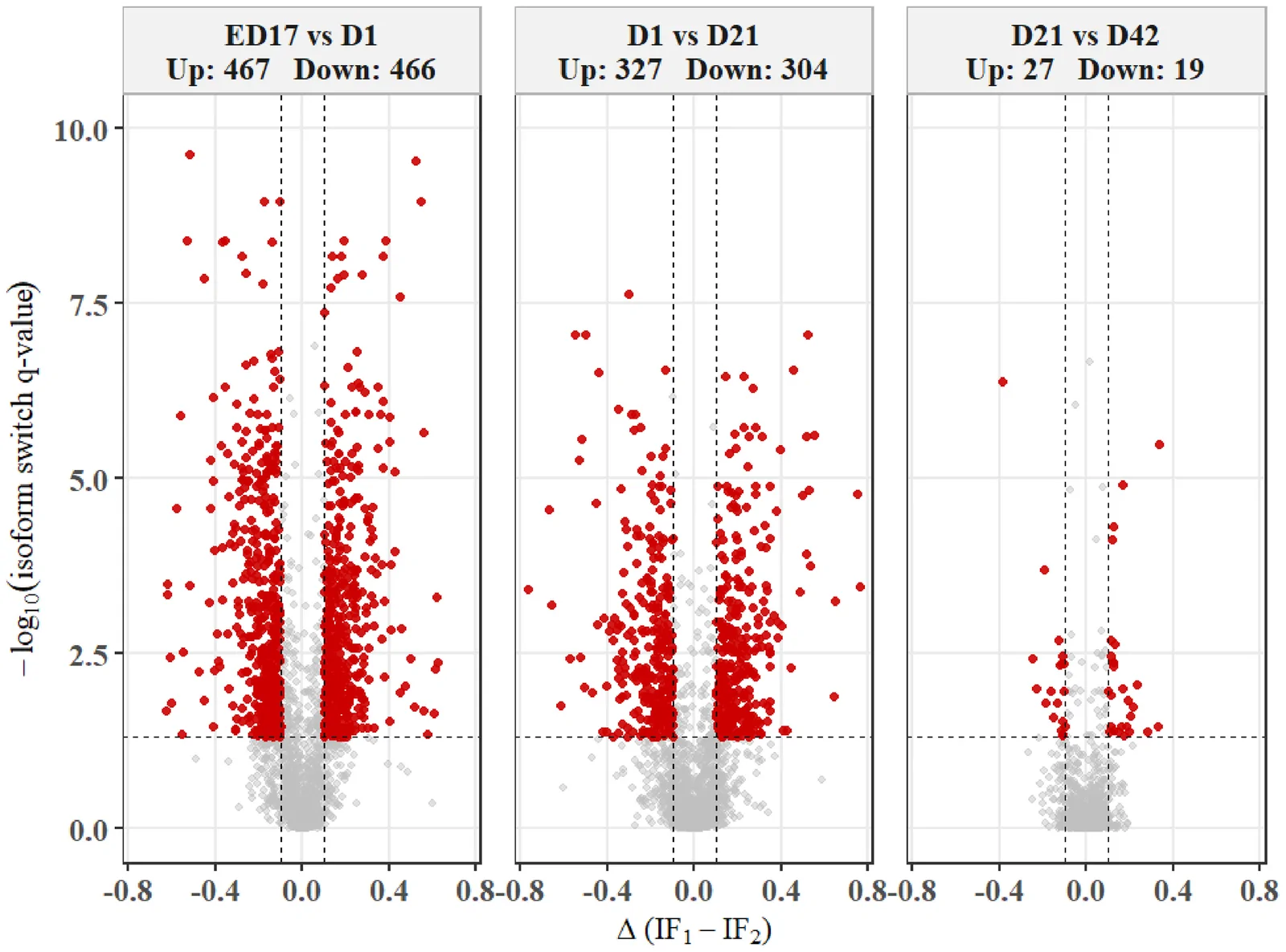
Volcano plots of isoform switching across developmental transitions in the breast muscles. Each point represents an isoform tested for differential usage between consecutive developmental stages: ED17 vs D1, D1 vs D21, and D21 vs D42. The *x*-axis shows the difference in isoform fraction between the two stages (ΔIF_1_-IF_2_), and the *y*-axis shows the -log_10_-transformed isoform switch *q*-value. Red points indicate significantly switched isoforms based on |Δ isoform fraction| > 0.1 and *q*-value < 0.05, whereas grey points indicate non-significant isoforms. Dashed vertical and horizontal lines indicate the ΔIF and significance thresholds, respectively. The number of isoforms with increased (Up) or decreased (Down) usage in each transition is shown above each panel.

Differential transcript usage analysis identified 933, 631, and 46 significant isoform switches across the ED17-to-D1, D1-to-D21, and D21-to-D42 developmental transitions, respectively (Fig. 2). During the ED17-to-D1 transition, 467 isoforms showed increased usage, whereas 466 isoforms showed decreased usage. During the D1-to-D21 transition, 327 isoforms increased in usage and 304 decreased in usage. In contrast, the D21-to-D42 transition showed markedly fewer significant switches, with only 27 isoforms increasing and 19 isoforms decreasing in usage.

**Fig. 3:**
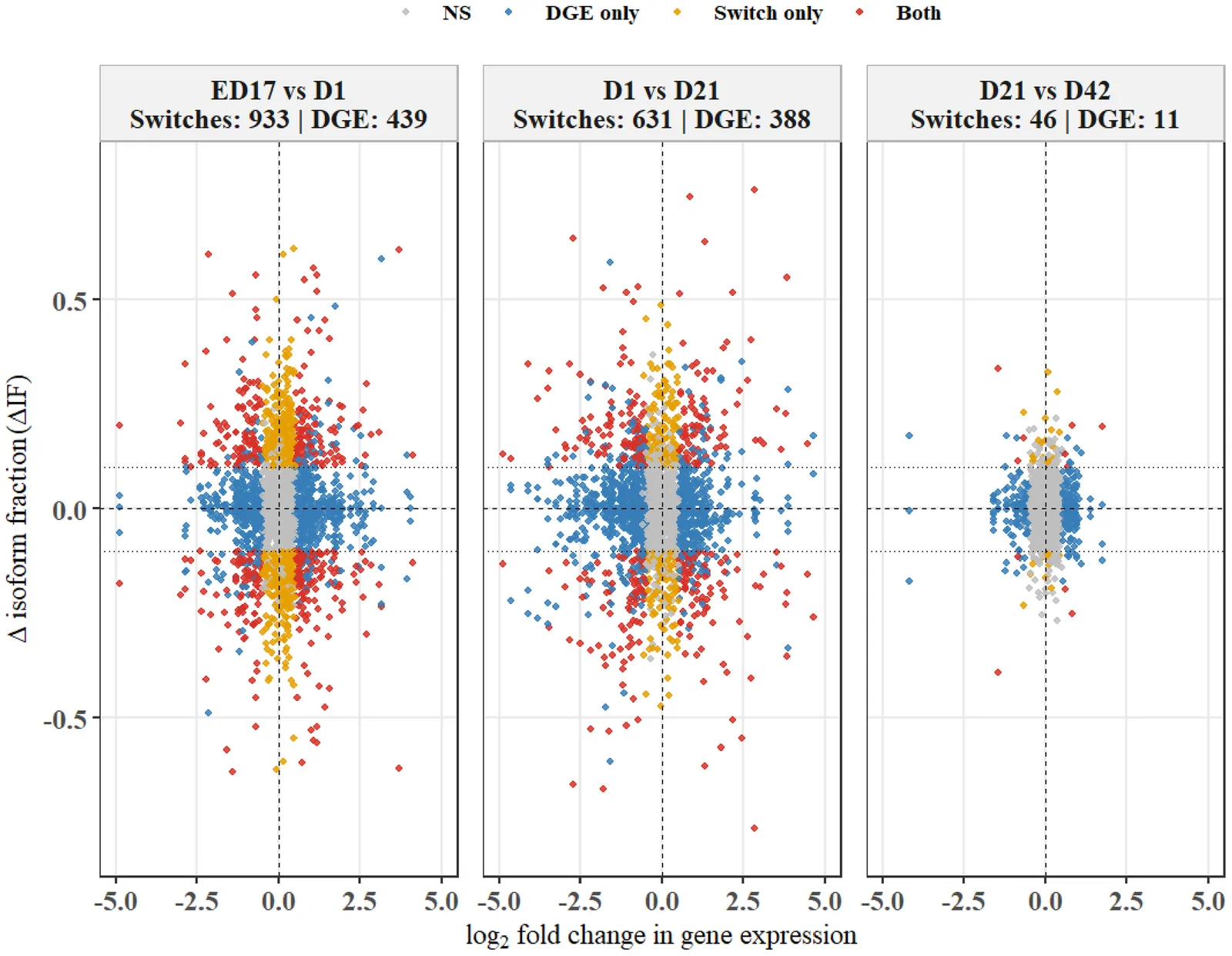
Relationship between gene-level expression changes and isoform usage changes across developmental transitions of the breast muscle. Each point represents an isoform mapped to its corresponding gene for the ED17-to-D1, D1-to-D21, and D21-to-D42 comparisons. The *x*-axis shows the gene-level log_2_ fold change in expression, and the *y*-axis shows the change in isoform fraction (ΔIF) between the two developmental stages. Isoforms are classified as non-significant (NS), associated with differentially expressed genes only (DGE only), significantly switched only (Switch only), or both differentially expressed and significantly switched (Both). Dashed vertical and horizontal lines indicate no change in gene expression and no change in isoform fraction, respectively, while dotted horizontal lines indicate the ΔIF threshold used to define isoform switching. The number of significantly switched isoforms (|Δ isoform fraction| > 0.1 and *q*-value < 0.05) and differentially expressed genes (|log_2_FC| > 0.5 & FDR < 0.05) detected in each transition is shown above each panel.

A substantial proportion of significantly switching isoforms occurred in genes that were not differentially expressed according to the predefined DGE criteria (Fig. 3). This represented 52.9%, 38.2%, and 71.7% of significant switches during the ED17-to-D1, D1-to-D21, and D21-to-D42 transitions, respectively.

**Fig. 4:**
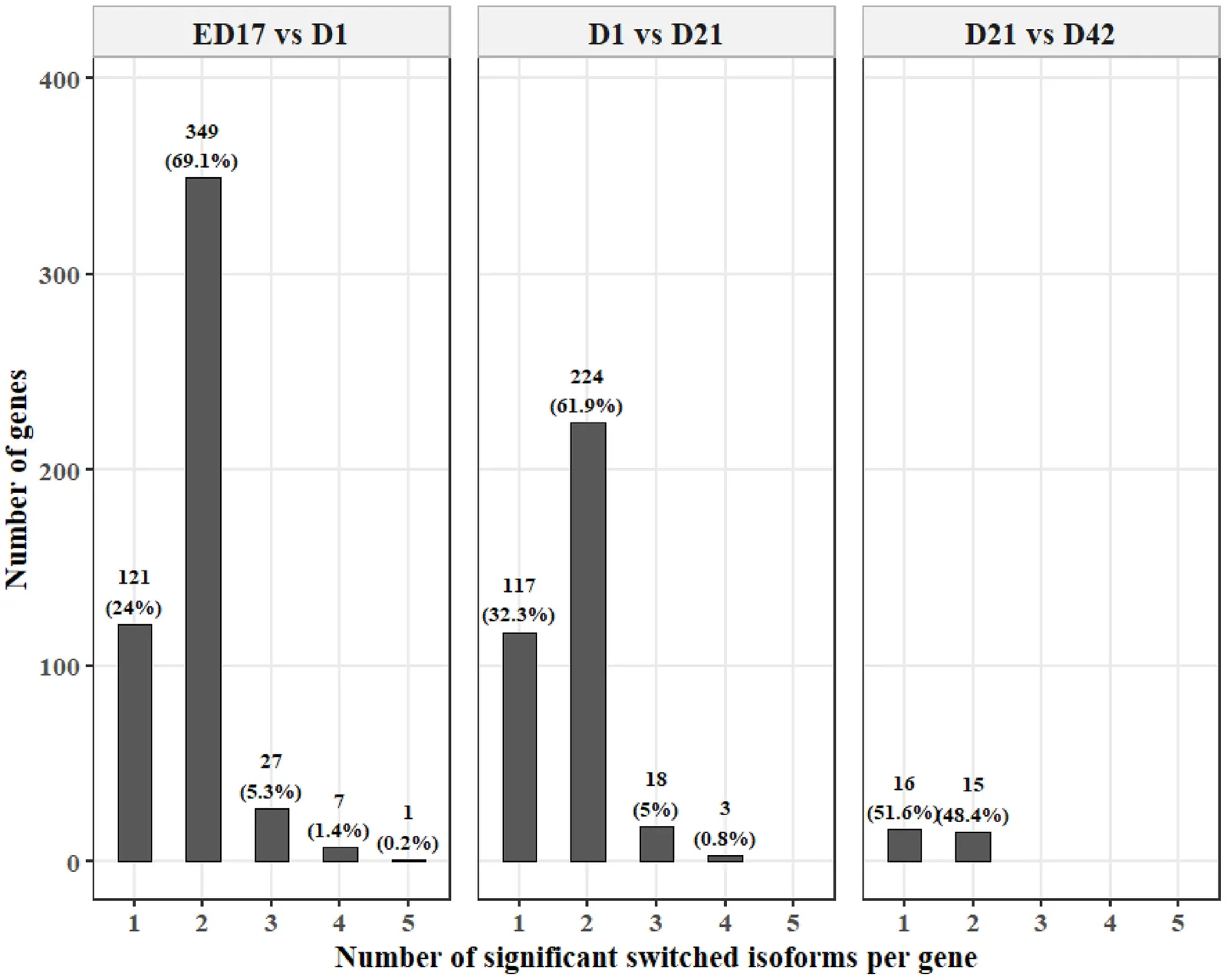
Distribution of the number of significantly switched isoforms per gene across developmental transitions in the breast muscle. Bars show the number of genes with one, two, or more significantly switched isoforms in each developmental comparison. Values above the bars indicate the number and percentage of switching genes within each transition.

Regarding the distribution of the number of isoforms involved in switching across developmental transitions, most significant switching genes had two significantly changing isoforms (Fig. 4).

### Predicted functional consequences of isoform switching

**Fig. 5:**
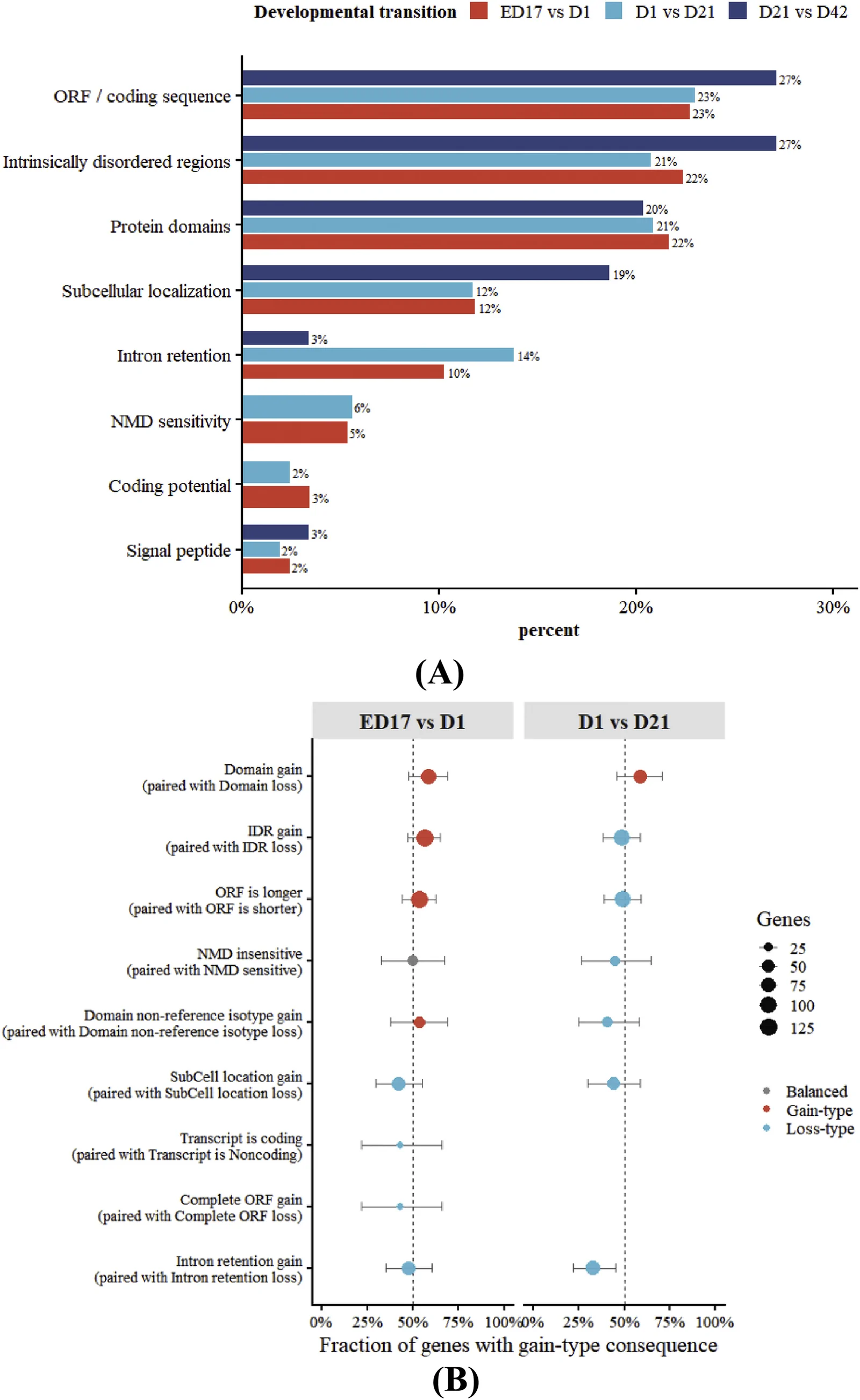
Predicted consequences of isoform switching in breast muscle. (A) Distribution of predicted consequence categories across developmental transitions in the breast muscle. Bars represent the percentage of predicted consequences assigned to each category within each transition. (B) Enrichment analysis of gain-type and loss-type consequences for selected consequence categories. Points represent the fraction of genes showing a gain-type consequence, and error bars indicate confidence intervals. Confidence intervals overlapping 50% indicate no significant deviation from an equal gain/loss balance.

The majority of significant switches were associated with predicted functional consequences at the protein level. Switches with predicted consequences represented 67%, 70%, and 76% of significant switches during the ED17-to-D1, D1-to-D21, and D21-to-D42 transitions, respectively. Across the three developmental transitions, the most frequent predicted consequence categories were changes affecting the ORF/coding sequence, intrinsically disordered regions, protein domains, and subcellular localization (Fig. 5A). These categories were relatively consistent across developmental stages. In contrast, predicted changes in nonsense-mediated decay (NMD) sensitivity, coding potential, and signal peptides were less frequent. Intron retention-associated consequences were more common during the ED17-to-D1 and D1-to-D21 transitions than during the D21-to-D42 transition, although the latter comparison included substantially fewer significant switches overall.

Consequence enrichment analysis showed that gain-type and loss-type consequences were generally balanced across developmental transitions (Fig. 5B). Most consequence categories had gain-type fractions close to 50%. In addition, enrichment comparisons across transitions did not identify significant differences in the enrichment of specific consequence categories.

### Alternative splicing (AS) patterns associated with isoform switching

**Fig. 6:**
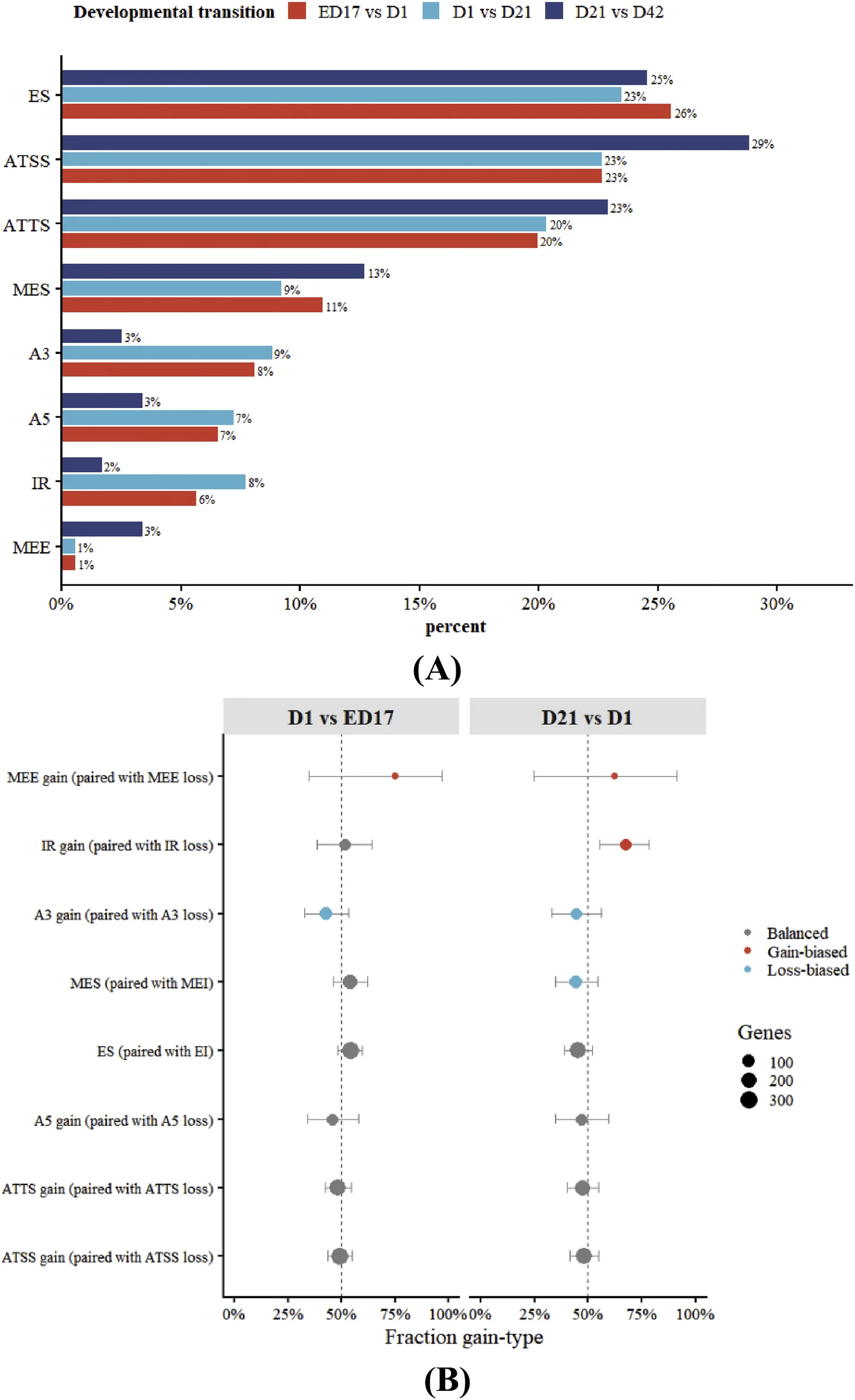
Alternative splicing patterns underlying isoform switching across developmental transitions in the breast muscle. (A) Distribution of alternative splicing event types across developmental transitions. Bars represent the percentage of each event type within each transition. (B) Enrichment analysis of gain-type and loss-type alternative splicing events. Points represent the fraction of genes showing a gain-type event, and error bars indicate confidence intervals. Confidence intervals overlapping 50% indicate no significant deviation from an equal gain/loss balance.

The analysis of alternative splicing patterns showed that exon skipping (ES), alternative transcription start sites (ATSS), and alternative transcription termination sites (ATTS) were the most represented event classes associated with significant isoform switches (Fig. 6A). These three classes were consistently among the most frequent across the three developmental transitions, with ATSS and ATTS showing a slight increase in relative frequency during the D21-to-D42 transition. Other events, including multiple exons skipping (MES), alternative 3′ splice sites (A3), alternative 5′ splice sites (A5), intron retention (IR), and mutually exclusive exons (MEE), were less frequent.

Alternative splicing enrichment analysis showed that gain-type and loss-type events were generally balanced across developmental transitions (Fig. 6B). Most splicing classes had gain-type fractions close to 50%. In addition, enrichment comparisons across developmental transitions did not identify significant differences in the representation of specific splicing classes.

### GO enrichment analysis

**Fig. 7:**
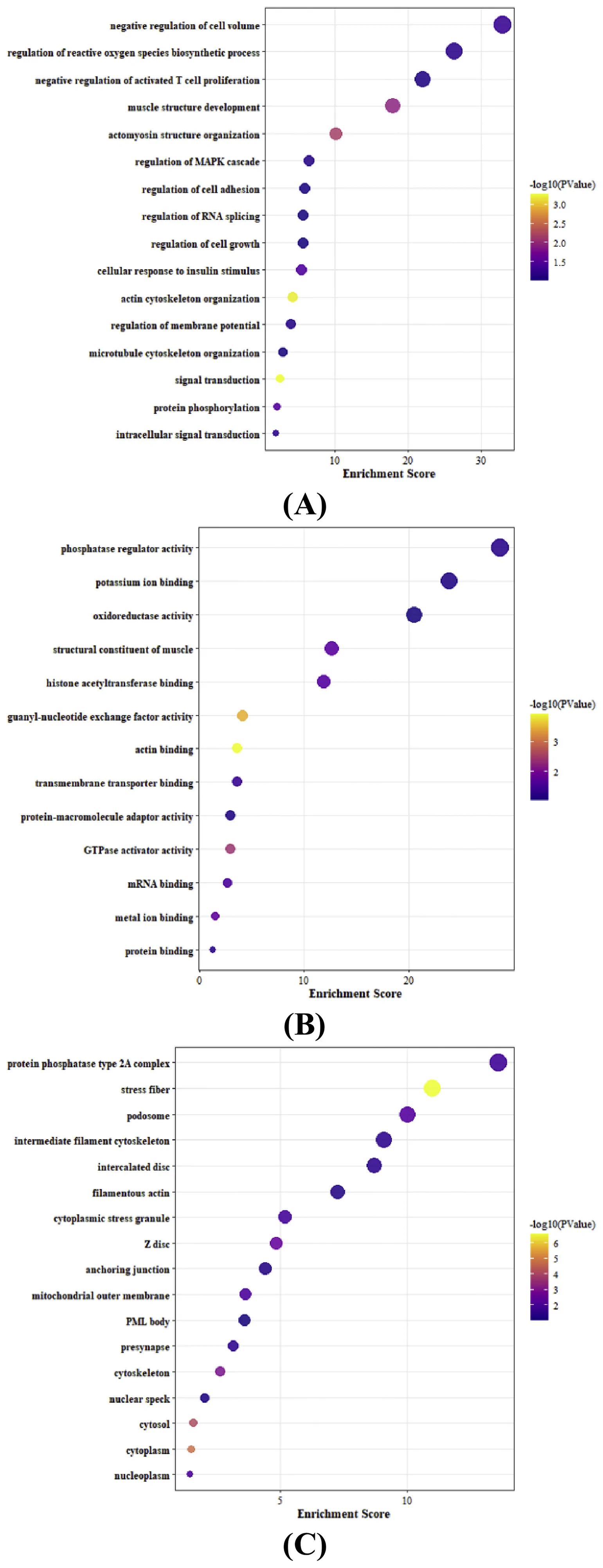
Gene ontology (GO) enrichment analysis of genes showing significant isoform switching during the ED17-to-D1 developmental transition in breast muscle. Panels show enriched GO terms for (A) biological processes, (B) molecular functions, and (C) cellular components among genes with significant isoform switching.

The results of the functional enrichment analysis of genes showing significant isoform switching between ED17 and D1 are presented in Fig. 7 and Supplementary Table S1. Enriched (*P* < 0.05) biological processes (Fig. 7A) included muscle structure development, actomyosin structure organization, cytoskeletal organization, cell adhesion, MAPK signaling, insulin response, RNA splicing regulation, and reactive oxygen species regulation. Molecular function terms (Fig. 7B) highlighted structural constituent of muscle, actin binding, oxidoreductase activity, phosphatase regulator activity, and small GTPase-related regulatory functions (*P* < 0.05). Cellular component enrichment (Fig. 7C) included (*P* < 0.05) stress fibers, filamentous actin, intermediate filament cytoskeleton, Z disc, anchoring junctions, and nuclear speckles.

**Fig. 8:**
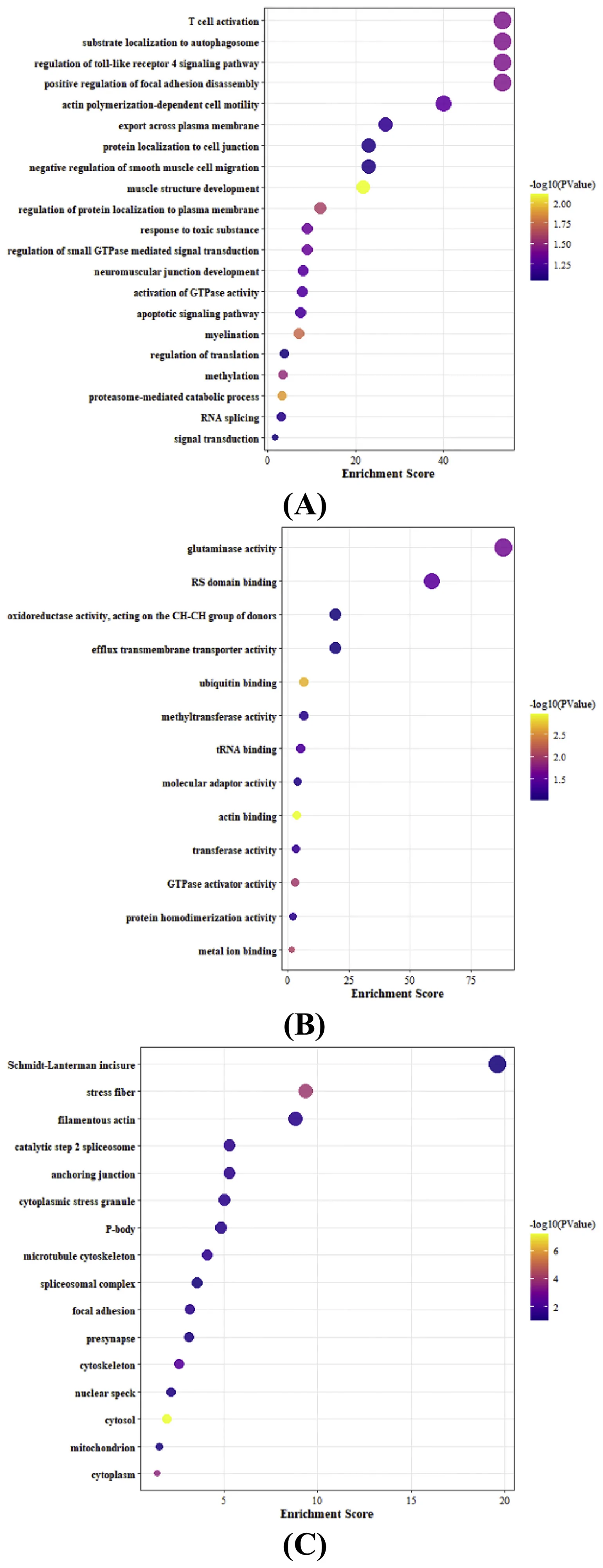
Gene ontology (GO) enrichment analysis of genes showing significant isoform switching during the D1-to-D21 developmental transition in breast muscle. Panels show enriched GO terms for (A) biological processes, (B) molecular functions, and (C) cellular components among genes with significant isoform switching.

As for the second developmental transition, enrichment analysis of genes showing significant isoform switching between D1 and D21 are shown in Fig. 8 and Supplementary Table S2. Biological process terms (Fig. 8A) included (*P* < 0.05) muscle structure development, focal adhesion disassembly, actin polymerization-dependent cell motility, neuromuscular junction development, GTPase-mediated signaling, RNA splicing, translational regulation, proteasome-mediated catabolism, and autophagosome-related processes. Molecular function terms (Fig. 8B) further highlighted RS domain binding, actin binding, GTPase activator activity, ubiquitin binding, glutaminase activity, methyltransferase activity, and oxidoreductase activity. Cellular component enrichment (Fig. 8C) included stress fibers, filamentous actin, focal adhesions, anchoring junctions, and microtubule cytoskeleton, together with RNA-processing compartments such as spliceosomal complexes, nuclear speckles, P-bodies, and cytoplasmic stress granules.

Given the small number of genes showing differential transcript usage during the D21-to-D42 transition, no enrichment analysis was conducted on these genes.

### Top-ranked isoform-switching genes across developmental transitions

**Fig. 9:**
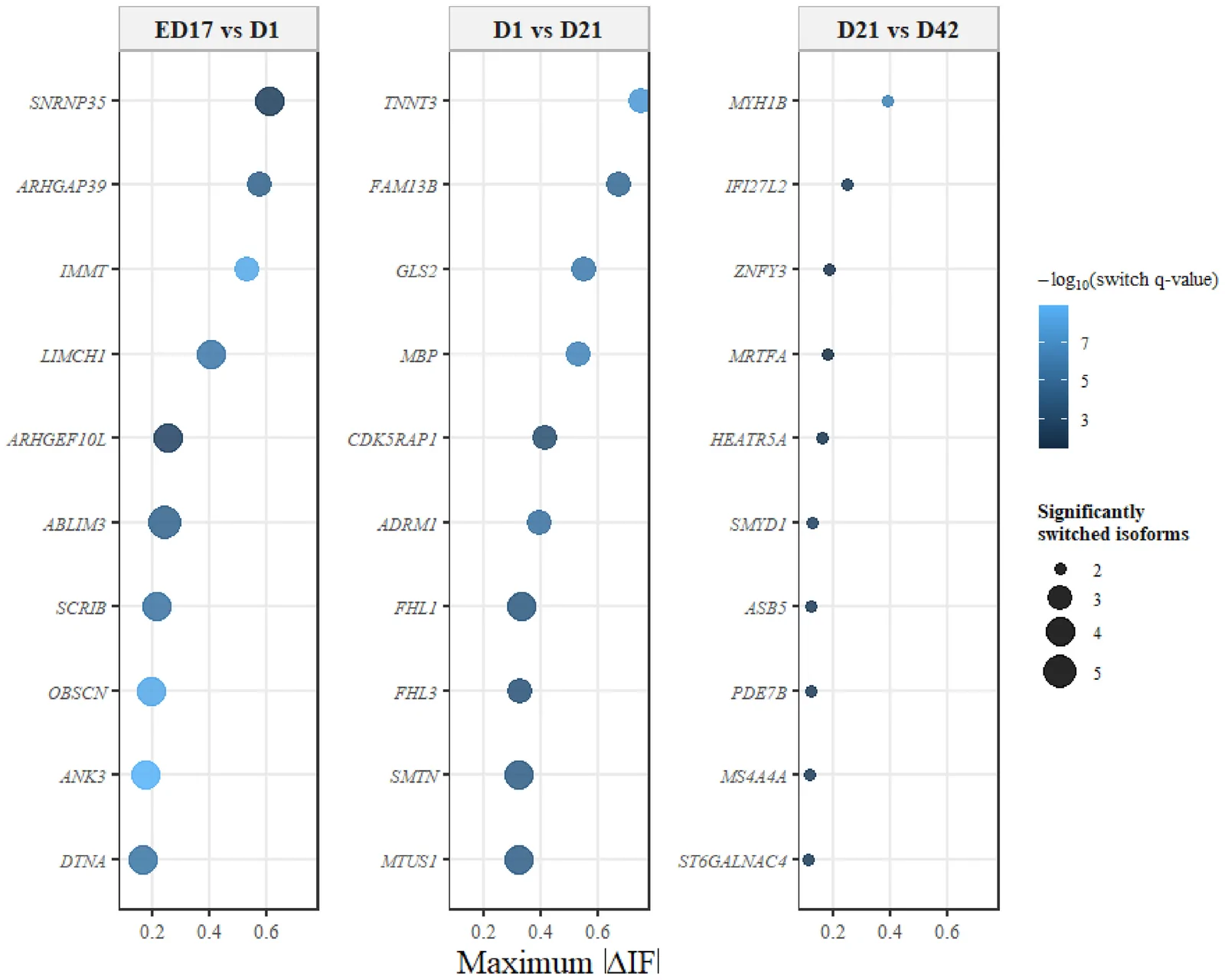
Top 10 genes showing the strongest differential transcript usage across breast muscle developmental transitions. Significant switching genes were ranked within each developmental transition according to the number of significantly switched isoforms, maximum absolute ΔIF, and minimum isoform switch *q*-value. Rankings are shown separately for each developmental comparison.

To identify genes showing the strongest differential transcript usage, significant switching genes were ranked within each developmental transition according to the number of significantly switched isoforms, maximum absolute ΔIF, and minimum switch *q*-value (Supplementary Table S3). Fig. 9 shows the top 10 ranked switching genes for each developmental transition. During the ED17-to-D1 transition, several top-ranked genes were associated with cytoskeletal or muscle structural organization, including OBSCN, DTNA, ABLIM3, and LIMCH1. During the D1-to-D21 transition, top-ranked switching genes included muscle-related genes such as TNNT3, FHL1, and FHL3. During the D21-to-D42 transition, genes with known relevance to contractile or muscle regulatory processes included MYH1B, SMYD1, and MRTFA.

## Discussion

Intensive genetic selection for increased growth rate and breast meat yield in broiler chickens has been associated with structural and metabolic perturbations in the *Pectoralis major* muscle (Soglia et al., 2020; Velleman, 2019). These perturbations are considered major contributors to breast muscle myopathies such as wooden breast and white striping (Alnahhas et al., 2023; Petracci et al., 2019). Although substantial progress has been made in understanding the mechanisms underlying these myopathies and other meat quality defects, their triggering factors remain incompletely understood, and effective mitigation strategies are still limited. Therefore, further characterization of normal breast muscle growth and development is needed to better understand these abnormalities.

RNA-Seq has been widely used to investigate muscle transcriptional regulation, biology, and pathology through gene-level analyses such as differential gene expression (Achour et al., 2025; Bordini et al., 2022; Brothers et al., 2019; Huang et al., 2025; Malila et al., 2019). However, gene-level analyses provide only a partial view of transcriptional regulation because multi-exon genes in higher eukaryotes can generate multiple transcript isoforms through alternative splicing and/or the use of alternative transcription start or termination sites (Froussios et al., 2019). In chicken gastrocnemius muscle, 37% of expressed genes were reported to undergo alternative splicing, with several top alternatively spliced genes involved in muscle development (Li et al., 2018). Similarly, satellite cells isolated from turkey breast muscle and exposed to thermal stress were predicted to show 61,266 splicing events across 5,202 annotated genes (Powell et al., 2026). Because transcript isoforms can differ in coding potential, regulatory features, stability, localization, and functional output, transcript-level analyses may reveal regulatory mechanisms that remain hidden in gene-level analyses (Cmero et al., 2019). Therefore, this study aimed to investigate the extent of gene isoform switching during broiler breast muscle development, predict its sequence- and protein-level consequences, and characterize the alternative splicing patterns associated with these switches.

### A shared developmental trajectory across genetic backgrounds

The dataset used in this study included three genetic backgrounds: Cornish, White Plymouth Rock, and their crossbred progeny. Although these three genetic groups differed in body weight and breast muscle mass (Huang et al., 2025), PCA based on normalized transcript counts indicated that developmental stage, rather than genetic background, was the main driver of transcriptomic variation in breast muscle. The three genetic groups followed a broadly similar developmental trajectory, consistent with the gene-level analysis of this dataset reported by Huang et al. (2025). Samples from different developmental time points formed clear clusters, with the embryonic stage separating from post-hatch stages and early post-hatch growth separating from later growth. This pattern is consistent with previous studies showing substantial transcriptomic changes and maturation of skeletal muscle across embryonic and post-hatch development (Jin et al., 2026; Wang et al., 2023). Therefore, downstream analyses were performed according to developmental stage while including samples from all three genetic backgrounds.

Interestingly, 52.9%, 38.2%, and 71.7% of significant switches during the ED17-to-D1, D1-to-D21, and D21-to-D42 transitions were found in genes that were not DEGs, respectively. These results indicate that considerable transcript-usage changes can occur even when overall gene expression changes are modest, highlighting the added resolution provided by DTU analysis compared with gene-level expression analysis alone (Froussios et al., 2019).

### Differential transcript usage (DTU) analysis

The initial dataset included 16,443 genes. After filtering to remove lowly expressed genes, for which isoform fraction estimates may be less precise (Vitting-Seerup and Sandelin, 2019), 7,602 genes were retained for further analysis, corresponding to 46.2% of the initial gene set. Across the three developmental transitions, 742 unique genes showed statistically significant evidence of isoform switching, defined as |ΔIF| > 0.1 and FDR < 0.05. Of these, 504 showed significant switching during ED17-to-D1, 361 during D1-to-D21, and 31 during D21-to-D42. It is worth noting that because some genes switched isoforms in more than one transition, these counts overlap and therefore do not sum to the total number of unique switching genes reported above. After further filtering for switches predicted to have at least one protein-level consequence, 498 unique genes remained, including 311, 232, and 19 overlapping genes in the ED17-to-D1, D1-to-D21, and D21-to-D42 transitions, respectively. This progressive decrease in the number of switching genes suggests that isoform-level remodeling is most pronounced around hatch and during early post-hatch growth, when breast muscle undergoes major structural, metabolic, and regulatory maturation (Givisiez et al., 2020). In contrast, the limited number of switching genes during the D21-to-D42 transition is consistent with a more advanced developmental stage in which the transcriptional and isoform-usage profiles are comparatively more stable (Jin et al., 2026; Wang et al., 2023). This pattern parallels the decrease in the number of significant DEGs observed during later growth phases (Huang et al., 2025; Liu et al., 2019), supporting the idea that both gene-expression and transcript-usage reprogramming decline as muscle development progresses.

During the ED17-to-D1 and D1-to-D21 transitions, most switched genes showed a limited number of significantly switched isoforms, with two significantly switched isoforms detected in 69.1% and 61.9% of switched genes, respectively. Genes with three or more significantly switched isoforms were less frequent, suggesting that most detected switching events involved relatively simple redistribution between a small number of transcript isoforms rather than complex multi-isoform changes. Although the D21-to-D42 transition included fewer switched genes overall, the same general tendency was observed, with most genes having one or two significantly switched isoforms. This pattern is consistent with the findings from Wang et al. (2023), who performed differential transcript expression (DTE) analysis of breast muscle from fast- and slow-growing chickens across multiple developmental time points and reported that multi-isoform genes often display stage-specific dominance of individual isoforms. Together, these results suggest that developmental transcript changes in breast muscle frequently involve shifts in the relative dominance of a limited number of isoforms within each gene.

### Patterns of alternative splicing driving protein-level consequences of isoform switching

Differential transcript usage reflects changes in the relative abundance of transcript isoforms and can arise from alternative splicing events, such as exon skipping (ES), alternative 5’ or 3’ splice-site usage (A5 or A3), and intron retention (IR), as well as from alternative transcription start site (ATSS) or alternative transcription termination site (ATTS) usage (Jiang and Chen, 2021; Powell et al., 2026). Therefore, isoform switching in this study was interpreted as transcript-isoform remodeling rather than exclusively as classical alternative splicing.

Among switched isoforms predicted to have protein-level consequences, ES was the most frequently detected event type across developmental transitions, with 1,042 events, followed by ATSS and ATTS, with 964 and 853 events, respectively. Although the absolute number of events decreased markedly with age, reflecting the lower number of switched isoforms during the D21-to-D42 transition, the relative distribution of event types was broadly conserved across transitions. ES, ATSS, and ATTS together accounted for the largest proportion of predicted events in all comparisons, whereas mutually exclusive exon usage, alternative splice-site usage, and intron retention were less frequent. This pattern indicates that developmental isoform switching in broiler breast muscle is driven mainly by exon skipping and alternative transcript boundary usage, rather than by complex mutually exclusive exon or intron-retention events. This is consistent with previous reports in mammals, where ES is a major mechanism contributing to transcript and protein diversity (Florea et al., 2013; Wang et al., 2017), and with studies in avian muscle. Wang et al. (2023) reported that ES was the most frequent AS type in chicken breast muscle from fast- and slow-growing strains, while Powell et al. (2026) observed that ES, ATSS, and ATTS were among the most common transcript-structure changes in turkey breast muscle satellite cells. Together, these findings suggest that exon skipping and alternative transcript start or termination site usage are conserved and prominent mechanisms contributing to developmental transcript diversification in avian skeletal muscle.

Gain- and loss-type AS events were defined according to whether the isoform increasing in usage during a developmental transition gained or lost a given transcript-structure feature relative to the isoform decreasing in usage. Across the ED17-to-D1 and D1-to-D21 transitions, for the most frequent event classes, including ES, ATSS, and ATTS, gain- and loss-type events did not significantly deviate from 50% and did not differ across developmental stages, indicating little global directional bias in these events. Thus, although these event types were common, their direction appeared gene-specific rather than uniformly associated with development.

In eukaryotes, most protein-coding genes contain introns and undergo pre-mRNA splicing, and a large proportion of multi-exon genes produce multiple transcript isoforms through alternative splicing (Tazi et al., 2009). Alternative splicing expands transcript and protein diversity, but its regulation must remain tightly controlled, as disruption of splicing programs has been associated with multiple diseases and developmental abnormalities (Kim et al., 2018; Tao et al., 2024). In the present study, the broadly balanced distribution between gain- and loss-type AS events may therefore reflect the regulated nature of isoform switching in normal developing breast muscle. Rather than indicating a unidirectional increase or decrease in specific AS features, this balance suggests that developmental transcript switching involves gene-wise redistribution of isoform structures, consistent with fine-tuning of transcript and protein diversity during muscle maturation.

### Predicted functional consequences of isoform switching

The AS patterns described above were associated with multiple predicted transcript- and protein-level consequences. The most common categories included changes in ORF/coding sequence architecture, including ORF length or completeness, which were detected in 497 switched isoforms, followed by changes in intrinsically disordered regions (*n* = 472 isoforms) and protein domains (*n* = 461 isoforms). These predicted consequences suggest that isoform switching may restructure protein architecture at multiple functional levels during breast muscle development. Changes in ORF length or completeness indicate alterations in the coding architecture of switched transcripts, which may generate protein isoforms differing in length, termini, or completeness (Bartonek et al., 2020; Meregalli et al., 2016). These ORF-level changes can then propagate to the protein level by altering their structured domains (PD) or intrinsically disordered regions (IDR). Protein domains generally represent folded functional modules involved in binding, catalysis, localization, or structural assembly, whereas intrinsically disordered regions provide flexible regulatory interfaces that can mediate transient interactions, post-translational modification, conformational plasticity, and multivalent binding (Langstein-Skora et al., 2026; Trivedi and Nagarajaram, 2022). This distinction between PDs and IDRs is particularly relevant in muscle, where sarcomeric proteins often combine structured domains with flexible disordered regions to regulate filament assembly, actomyosin interaction, contractile tuning, and mechanosensitive regulation (Tolkatchev et al., 2019). Moreover, alternatively spliced coding regions can generate protein segments enriched in intrinsic disorder, suggesting that transcript-isoform changes may create or remove flexible functional regions with regulatory potential (Kovacs et al., 2010). Therefore, the frequent prediction of ORF/coding sequence, IDR, and PD consequences in the present study suggests that developmental isoform switching may fine-tune both the structural modules and regulatory flexibility of proteins involved in breast muscle maturation and growth.

Predicted changes in subcellular localization of proteins were also observed (*n* = 259 isoforms), mainly during the ED17-to-D1 and D1-to-D21 transitions. These changes frequently involved transitions between annotated compartments and isoforms with no predicted localization, suggesting that isoform switching may alter the presence of recognizable targeting features of proteins. The most recurrent predicted localization changes involved nuclear, cytoplasmic, mitochondrial, and cell-membrane-associated annotations. Although these predictions require cautious interpretation, they suggest that developmental isoform switching may influence not only protein structure (*i.e.,* PDs and IDRs) but also the compartmental context in which proteins act during breast muscle maturation.

Changes in intron retention status were also predicted among switched isoforms (n = 252 isoforms), especially during the ED17-to-D1 (n = 119 isoforms) and D1-to-D21 (n = 111 isoforms) transitions. Intron retention can influence transcript fate by altering ORF structure, introducing premature termination codons, promoting nuclear retention, or reducing productive protein output. Its relevance to muscle biology is supported by studies showing that intron retention acts as a regulated post-transcriptional mechanism during skeletal muscle stem cell quiescence exit (Yue et al., 2020) and that altered intron-retention profiles are associated with protein-expression changes in muscle disease contexts (Xiao et al., 2024). Thus, intron-retention changes in the present study may represent an additional mechanism through which isoform switching modulates transcript fate during breast muscle development.

Other consequence categories, including changes in nonsense-mediated decay (NMD) sensitivity and in coding potential, were detected in the present study at lower frequencies. Together, these results suggest that developmental isoform switching in broiler breast muscle may alter both coding-region structure and protein features, with potential effects on protein length, domain composition, structural flexibility, localization, and transcript stability.

Directional analysis of predicted isoform-switch consequences did not reveal significant deviations from the expected 50% gain/loss distribution for most consequence categories, indicating that gain- and loss-type consequences were broadly balanced across breast muscle developmental transitions. This suggests that developmental isoform switching does not produce a uniform shift toward gain or loss of specific protein features but rather reflects gene-specific alterations of transcript and protein architecture. This pattern is consistent with the absence of strong directional enrichment for specific AS event types described in the previous section.

### GO enrichment analysis of switching genes

***ED17 to D1 transition***. Functional enrichment analysis of genes showing significant isoform switching between ED17 and D1 revealed a predominant signature of structural, metabolic, and regulatory reprogramming in breast muscle. This is consistent with the biological context of the hatch transition, during which the embryo shifts from an *in ovo* environment dependent mainly on yolk-derived nutrients to a post-hatch state requiring pulmonary respiration, thermoregulation, movement, feeding, and increased metabolic activity (Givisiez et al., 2020). Accordingly, enriched biological processes included muscle structure development, actomyosin structure organization, actin cytoskeleton organization, regulation of cell adhesion, and regulation of cell growth, suggesting that isoform switching during this transition affected genes involved in the organization and maturation of the muscle structural machinery.

This interpretation was further supported by cellular component terms such as stress fiber, filamentous actin, intermediate filament cytoskeleton, cytoskeleton, anchoring junction, and Z disc, together with molecular functions such as structural constituent of muscle and actin binding. These terms indicate that isoform switching was associated with genes involved in cytoskeletal organization, sarcomeric architecture, and mechanical stabilization of muscle cells. The enrichment of the Z disc term is particularly relevant because the Z disc is central to sarcomere organization and structural integrity (Ahn et al., 1993; Luther, 2009). Beyond structural remodeling, enrichment of biological processes related to regulation of reactive oxygen species biosynthesis and molecular functions such as oxidoreductase activity may reflect isoform-level regulation of genes involved in redox homeostasis and metabolic adaptation, consistent with the increased oxygen exposure and mitochondrial activity that accompany the onset of pulmonary respiration at hatch (De Oliveira et al., 2008; Givisiez et al., 2020).

Several enriched terms also pointed to regulatory changes at the RNA, signaling, and chromatin levels. The enrichment of regulation of RNA splicing, together with nuclear speck components, suggests that some switches occurred in genes involved in RNA processing. Notably, this category included MBNL1, MBNL3, and CLK4, which are known regulators of alternative splicing in muscle tissue (Huang et al., 2022; Lee et al., 2010; Yadava et al., 2024), suggesting that isoform switching may affect splicing regulators themselves and potentially contribute to broader transcriptomic reprogramming during the hatch transition. Enrichment of guanyl-nucleotide exchange factor activity and GTPase activator activity further suggests involvement of small GTPase signaling, which regulates cytoskeletal dynamics, membrane trafficking, cell shape, adhesion, and migration (Bryan et al., 2005), processes relevant to myoblast organization, myotube maturation, sarcomere assembly, and muscle cell architecture (Rodríguez-Fdez and Bustelo, 2021).

Finally, enrichment of histone acetyltransferase binding suggests that isoform switching may also affect genes connected to chromatin-level transcriptional control. This is biologically plausible because the ED17-to-D1 transition is associated with major transcriptional reprogramming of breast muscle (Liu et al., 2019; Wang et al., 2023), and histone acetylation is a key post-translational modification involved in chromatin dynamics and transcriptional regulation (McCullough and Marmorstein, 2016). One switching gene enriched in this category was KANSL1, a component of the non-specific lethal complex, which regulates chromatin-associated transcriptional networks important for developmental processes (Sheikh et al., 2019). In addition, enrichment of cytoplasmic stress granule components may reflect post-transcriptional adaptation to hatch-associated physiological stress, since stress granules sequester translationally inactive mRNAs during cellular stress and contribute to translational control (Ivanov et al., 2019).

Overall, the ED17-to-D1 enrichment profile suggests that isoform switching is functionally biased toward genes involved in sarcomeric and cytoskeletal organization, redox and metabolic adaptation, signaling, chromatin regulation, and RNA-processing control. These results support the view that the hatch transition is accompanied not only by changes in gene expression, but also by isoform-level remodeling of genes associated with the structural and regulatory maturation of breast muscle. Importantly, during this transition, the effects of chronological age cannot be clearly separated from those of the hatch-associated environmental and physiological changes. The observed isoform-switching patterns should therefore be interpreted as reflecting the combined influence of intrinsic age-dependent muscle maturation and adaptation from the pre-hatch to the post-hatch environment.

***D1 to D21 transition***. Between D1 and D21, significant isoform switching was enriched in genes involved in cytoskeletal organization, focal adhesion dynamics, RNA processing, translation control, protein turnover, autophagy, metabolic maturation, and immune/stress-related signaling. This profile is consistent with the biological context of this transition, during which broiler breast muscle undergoes rapid post-hatch growth through hypertrophy of existing fibers (Gu et al., 2024; Hoque et al., 2026). This growth phase is accompanied by extensive reorganization of cytoskeletal structure, cell-matrix interactions, mechanical loading, and neuromuscular maturation (Huang et al., 2025), suggesting that differential transcript usage may contribute to the structural and regulatory adaptations associated with rapid breast muscle growth.

A major feature of the D1-to-D21 enrichment profile was the involvement of cytoskeletal, adhesion, and small GTPase-related terms, including focal adhesion-related processes, actin polymerization-dependent cell motility, protein localization to cell junctions, actin binding, GTPase activator activity, stress fiber, filamentous actin, focal adhesion, anchoring junction, and microtubule cytoskeleton. Together, these terms suggest that isoform switching may affect genes involved in how muscle cells sense, attach to, and remodel their extracellular and cytoskeletal environment. This is biologically coherent with rapid hypertrophic growth, because expanding muscle fibers and their supporting connective structures must adapt mechanically as fiber size increases.

The enrichment profile also indicated extensive post-transcriptional and proteostatic regulation. Terms related to RNA splicing, RS domain binding, spliceosomal complex, catalytic step 2 spliceosome, nuclear speck, regulation of translation, tRNA binding, P-body, cytoplasmic stress granule, proteasome-mediated catabolic process, and substrate localization to autophagosome suggest that isoform switching during this period was associated with RNA processing, mRNA fate, translation control, protein degradation, and autophagy. The enrichment of RS domain binding is relevant because RS domains can function as splicing activation domains when tethered to pre-mRNA (Philipps et al., 2003). Overall, these terms suggest active reprogramming of the transcriptome and proteome, consistent with the coordinated protein synthesis, degradation, and renewal required during rapid muscle growth (Di Luca et al., 2024; Liu et al., 2016; Nakka et al., 2018; Sun et al., 2021).

Metabolic and stress-related terms further suggest that isoform switching may contribute to adaptation to the high energetic and physiological demands of early post-hatch growth. The enrichment of glutaminase activity is notable because glutamine metabolism contributes to energy balance, nucleotide synthesis, redox homeostasis, and anabolic growth (Hu et al., 2020; Liu et al., 2025). Since skeletal muscle is a major site of glutamine synthesis and release to the circulation, where it can support energy-producing pathways in other tissues (Smith et al., 1984), this enrichment may reflect isoform-level regulation of genes involved in metabolic adaptation during rapid growth. In parallel, immune/stress-response terms may reflect increasing metabolic demand and low-grade stress signaling associated with rapid broiler growth (Song et al., 2021; Tickle et al., 2018; Ulans et al., 2026), rather than a purely immune-specific response.

Overall, the D1-to-D21 enrichment profile suggests that isoform switching is associated with early post-hatch breast muscle growth through changes in genes involved in cytoskeletal restructuring, adhesion and mechanosensing, RNA-processing regulation, protein turnover, autophagy, metabolic adaptation, and stress-related signaling. In contrast to the ED17-to-D1 transition, which appeared to reflect hatch-associated adaptation from the embryonic to post-hatch phase, the D1-to-D21 transition seems more strongly associated with the structural, metabolic, and post-transcriptional regulation of early hypertrophic muscle growth.

### Examples of top-ranked isoform switches highlight candidate regulators of muscle maturation

Among the top genes showing significant isoform switching, several were directly related to muscle structure, function, and developmental regulation, with distinct patterns across developmental transitions. In the following section, key examples of these genes are discussed.

During ED17-to-D1, examples such as OBSCN and DTNA supported a role for isoform switching in sarcomeric and cytoskeletal remodeling around hatch. OBSCN encodes obscurin, a giant myocyte protein involved in sarcomere organization, myofibrillogenesis, elasticity, stretch response, and the structural integration of contractile and cytoskeletal elements (Di Paola and Schöck, 2026). Its selection among the top-ranked switching genes is therefore biologically consistent with the major changes of breast muscle that occur around hatch, when embryonic myofiber formation gives way to post-hatch fiber growth and increasing mechanical demand. In the present dataset (Supplementary Fig. S2), OBSCN expression was markedly higher at D1 than at ED17, indicating increased requirement for this sarcomeric/cytoskeletal scaffold during early post-hatch muscle maturation. Importantly, this transcriptional upregulation was accompanied by a redistribution of isoform usage. More specifically, three isoforms that together represented approximately half of the OBSCN transcript output at ED17 decreased after hatch, whereas two isoforms increased and together accounted for a large proportion of OBSCN expression at D1. This pattern suggests that the ED17-to-D1 transition involves replacement of embryonic OBSCN isoforms by post-hatch isoforms. Because the switched transcripts were predicted to be coding and cytoplasmic, the switch is unlikely to reflect a simple loss of OBSCN protein production or a major change in subcellular localization. Rather, the predicted loss of an intrinsically disordered region in the post-hatch isoforms suggests a possible restructuring of obscurin protein architecture, with potential consequences for its interaction with sarcomeric, cytoskeletal, or regulatory partners. Thus, OBSCN illustrates how isoform switching may contribute to breast muscle maturation by modifying the molecular form of a key structural protein while its overall expression increases.

The DTNA gene encodes α-dystrobrevin, a component of the macromolecular dystrophin-glycoprotein complex (DGC) that binds dystrophin/utrophin and α-syntrophin (Nascimento et al., 2023). The lack of α-dystrobrevin in mice has been associated with a muscular dystrophy phenotype (Nascimento et al., 2023), supporting its functional importance in maintaining muscle integrity. In the present study, DTNA was significantly upregulated in breast muscle from ED17 to D1 and showed marked redistribution of isoform usage during this transition (Supplementary Fig. S3). Among the five detected coding isoforms, four showed significant isoform switching, and all encoded proteins were predicted to localize to the cytoplasm. At ED17, two isoforms accounted for approximately half of the total DTNA transcript output, whereas after hatch, two alternative isoforms increased and together represented approximately 75% of DTNA expression at D1. This pattern suggests that the developmental increase in DTNA expression was driven by preferential use of post-hatch isoforms. Because α-dystrobrevin contributes to the DGC, this isoform redistribution may reflect maturation of the molecular link between the contractile apparatus, cytoskeleton, and sarcolemma during the transition to post-hatch muscle function. Notably, the post-hatch isoforms were longer than the embryonic isoforms and were predicted to differ in intrinsically disordered regions, suggesting potential differences in protein flexibility or interaction capacity. Therefore, DTNA provides an example in which isoform switching may alter the structural or regulatory properties of a key DGC-associated protein during early breast muscle maturation.

During D1-to-D21, genes such as TNNT3 and FHL1 illustrated isoform switching in contractile and mechanoregulatory genes during rapid post-hatch growth. In skeletal muscle, the troponin complex is composed of three regulatory proteins, troponin C, troponin I, and troponin T, and plays a central role in the regulation of muscle contraction (Ju et al., 2013). Troponin T3 (TNNT3) is a fast skeletal muscle troponin isoform (Altin et al., 2025) that is required for postnatal growth and survival in mice (Ju et al., 2013). In the present study, TNNT3 expression increased significantly in breast muscle from D1 to D21 and was accompanied by a pronounced redistribution of isoform usage (Supplementary Fig. S4). At D1, TNNT3 transcript output was distributed across several coding isoforms, whereas by D21, one isoform became strongly predominant and accounted for more than 75% of total TNNT3 expression. This pattern suggests that the post-hatch increase in TNNT3 expression involved developmental consolidation toward a specific TNNT3 transcript variant. All detected isoforms were predicted to be coding and to encode cytoplasmic proteins. Notably, the dominant D21 isoform was shorter than the other isoforms and was predicted to contain fewer intrinsically disordered regions while retaining the troponin domain. This switch may therefore reflect maturation of the fast-contractile apparatus in breast muscle, with isoform switching potentially refining the structural or regulatory properties of TNNT3 during early post-hatch growth.

The Four-and-a-half LIM domain protein 1 (FHL1) is highly present in skeletal muscle and plays important roles in protein synthesis, cellular integrity, and the intracellular Ras/mitogen-activated protein kinase pathway (Hu et al., 2019). Mutations in this gene are also known to cause myopathies in humans (Caputo and Schoser, 2024), supporting its relevance to muscle structure and function. In the present analysis, FHL1 had four detected isoforms in breast muscle, all predicted to be coding transcripts encoding cytoplasmic proteins (Supplementary Fig. S5). FHL1 expression was markedly downregulated from D1 to D21, but this decrease was accompanied by a clear redistribution of isoform usage. At D1, FHL1 transcript output was distributed mainly across multiple isoforms, whereas at D21 one shorter isoform became predominant, accounting for more than 60% of total FHL1 usage. A second isoform also increased in relative usage at D21, although to a lesser extent. It is important to note that because total FHL1 expression was strongly reduced, the increased usage of these D21-associated isoforms reflects their relative predominance within a reduced FHL1 transcript pool. At the protein level, the switched isoforms retained LIM-related domains, suggesting that the switch may not abolish the core domain architecture of FHL1 but may instead modify isoform structure and potentially its regulatory or interaction properties. Thus, FHL1 provides an example in which post-hatch muscle maturation is associated with both reduced expression of a muscle-associated regulatory gene and selective retention of specific isoforms during the D1-to-D21 transition.

During D21-to-D42, switches in genes such as MYH1B and SMYD1 suggested later fine-tuning of contractile phenotype and muscle regulatory programs. The Myosin Heavy Chain 1B (MYH1B) gene has been shown to reduce muscle cell proliferation and to increase myoblast differentiation in broiler muscles (Yu et al., 2021). In the present study, three MYH1B isoforms were detected in breast muscle, all predicted to be coding transcripts encoding proteins localized to the cytoplasm and/or membrane (Supplementary Fig. S6). MYH1B expression was significantly downregulated at D42 relative to D21, but this decrease was accompanied by a marked redistribution of isoform usage. At D21, one isoform accounted for approximately 80% of total MYH1B transcript output, whereas by D42 its usage decreased to around 40%. In contrast, a shorter isoform increased in relative usage and became the predominant transcript at D42, accounting for approximately 50% of MYH1B expression, while a third isoform also increased modestly in relative usage. Because total MYH1B expression declined substantially, the increased usage of the D42-associated isoforms reflects their relative predominance within a reduced MYH1B transcript pool, as mentioned previously. All three isoforms retained the predicted myosin head and myosin tail domains, suggesting that the switch may preserve the core motor-domain architecture while altering isoform length and potentially regulatory or structural features. Thus, MYH1B illustrates a late post-hatch switching pattern in which reduced expression of a growth-associated myosin gene is accompanied by replacement of a dominant D21 isoform by shorter D42-associated transcripts.

The SET and MYND domain containing 1 (SMYD1) gene encodes a muscle-specific histone methyltransferase that catalyzes lysine methylation on histone proteins and contributes to the regulation of gene expression, cell proliferation, and differentiation (Duan et al., 2026; Warren et al., 2018). In the present analysis, SMYD1 had two coding isoforms in breast muscle, and its overall expression was significantly downregulated from D21 to D42 (Supplementary Fig. S7). This decrease was accompanied by a significant redistribution of isoform usage. At D21, one isoform represented slightly more than 40% of total SMYD1 transcript output, whereas by D42 its usage decreased to approximately 30%. Conversely, the second isoform became predominant at D42, accounting for approximately 70% of total SMYD1 usage. Because total SMYD1 expression declined, this pattern reflects relative retention of the D42-dominant isoform within a reduced SMYD1 transcript pool. Both isoforms were predicted to encode cytoplasmic and nuclear proteins and to retain the MYND domain, but the D42-associated isoform was predicted to lack one SET domain relative to the D21-dominant isoform. This structural difference may be relevant because SET domains are directly linked to methyltransferase activity, whereas the MYND domain contributes to protein interactions and transcriptional regulation (Spellmon et al., 2015). Thus, SMYD1 illustrates a late post-hatch isoform switch in which reduced expression of a muscle-specific epigenetic regulator is accompanied by a shift toward an isoform with a potentially different catalytic or regulatory configuration.

Together, these examples suggest that isoform switching during breast muscle development affects both structural components and regulatory pathways associated with hatch-related maturation, rapid post-hatch growth, and later refinement of contractile function.

### Limitations and future directions

One limitation of this study is that the predicted consequences of isoform switching were inferred computationally and were not experimentally validated. Because this study used publicly available RNA-Seq data, the original biological samples were not available for additional validation experiments. Given the exploratory nature of the analysis, these predictions are useful for identifying candidate switches that may affect coding sequence, protein domains, intrinsically disordered regions, subcellular localization, or NMD sensitivity. However, they do not directly demonstrate changes at the protein or functional level. Therefore, the predicted consequences should be interpreted as hypotheses requiring validation by isoform-specific transcript assays, long-read sequencing, proteomics, localization studies, and functional experiments.

## Conclusions

This study characterized gene isoform switching in the breast muscle of meat-type chickens from late embryonic development to market age, including the associated alternative splicing patterns and their predicted sequence- and protein-level consequences. To our knowledge, this is the first extensive examination of isoform-level transcriptional regulation in chicken breast muscle. Developmental patterns were broadly consistent across genetic groups, with the largest wave of isoform switching occurring around hatch and progressively declining during post-hatch structural and metabolic maturation. The most frequently predicted consequences involved changes in ORF length, intrinsically disordered regions, and protein domains, mainly associated with exon skipping and alternative transcription start and termination sites. Genes with switched isoforms around hatch were enriched in functions related to the embryonic-to-post-hatch structural and metabolic transition, whereas those switching between hatch and three weeks of age were more closely associated with hypertrophic growth. These findings indicate that isoform switching provides an additional layer of transcriptomic regulation that may contribute to hatch-related maturation, rapid post-hatch growth, and later refinement of contractile function. Future studies should validate these findings using isoform-specific assays and long-read sequencing.

## Disclosures

The authors declare the following financial interests/personal relationships which may be considered as potential competing interests:

Nabeel Alnahhas reports financial support was provided by Natural Sciences and Engineering Research Council of Canada. If there are other authors, they declare that they have no known competing financial interests or personal relationships that could have appeared to influence the work reported in this paper.

## Appendix Supplementary materials

Image, application 1

Image, application 2

## Footnotes

Supplementary material associated with this article can be found, in the online version, at doi:10.1016/j.psj.2026.107583.
