# Supplementary material for: Transcript isoform switching during embryonic and post-hatch development of broiler breast muscle
Source: Poult Sci. 2026 Aug 5;105(11):107583. doi: 10.1016/j.psj.2026.107583 (PMC13521219; doi:10.1016/j.psj.2026.107583)
Supplement: Supplementary file 1 [file mmc1.docx]

**Transcript isoform switching during embryonic and post-hatch development of broiler breast muscle**

Nabeel Alnahhas^*, #,1^

^*^ Department of Animal Science, Faculty of Agricultural and Food Sciences, Université Laval, 2425 Rue de l’Agriculture, Quebec City G1V 0A6, Quebec, Canada

*^#^* Swine and Poultry Infectious Diseases Research Centre, Faculty of Veterinary Medicine, Université de Montréal, 3200 Rue Sicotte, St-Hyacinthe J2S 2M2, Quebec, Canada

^1^Corresponding author:

Nabeel Alnahhas

E-mail: [nabeel.alnahhas@fsaa.ulaval.ca](mailto:nabeel.alnahhas@fsaa.ulaval.ca)

**Supplementary Figure S1**

**Supplementary Figure S1.** Bioinformatics workflow for isoform switching analysis.

**Supplementary Figure S2**

**Supplementary Figure S2.** Isoform switch in OBSCN during the ED17-to-D1 developmental transition. The upper panel shows the transcript structure of the detected OBSCN isoforms, including predicted coding status, protein domains, and subcellular localization. The lower panels show total gene expression, isoform-level expression, and isoform usage, expressed as isoform fraction, at ED17 and D1. OBSCN expression was significantly upregulated from ED17 to D1, and this increase was accompanied by redistribution of isoform usage from ED17-associated isoforms toward D1-associated isoforms. All detected isoforms were predicted to be coding transcripts, with encoded proteins predicted to localize to the cytoplasm. Asterisks indicate significant differences between developmental stages.

**Supplementary Figure S3**

**Supplementary Figure S3.** Isoform switch in DTNA during the ED17-to-D1 developmental transition. The upper panel shows the transcript structure of the detected DTNA isoforms, including predicted coding status, protein domains, and subcellular localization. The lower panels show total gene expression, isoform-level expression, and isoform usage, expressed as isoform fraction, at ED17 and D1. DTNA expression was significantly upregulated from ED17 to D1, and this increase was accompanied by redistribution of isoform usage from ED17-associated isoforms toward D1-associated isoforms. All detected isoforms were predicted to be coding transcripts, with encoded proteins predicted to localize to the cytoplasm. Asterisks indicate significant differences between developmental stages.

**Supplementary Figure S4**

**Supplementary Figure S4.** Isoform switch in TNNT3 during the D1-to-D21 developmental transition. The upper panel shows transcript structure, predicted coding status, protein domains, and subcellular localization for the five detected TNNT3 isoforms. The lower panels show total gene expression, isoform-level expression, and isoform usage, expressed as isoform fraction, at D1 and D21. TNNT3 was significantly upregulated at D21, and this increase was accompanied by a marked shift in isoform usage, with ENSGALT00010065394.1 becoming the predominant isoform at D21. All detected isoforms were predicted to be coding transcripts encoding cytoplasmic proteins. Asterisks indicate significant differences between developmental stages.

**Supplementary Figure S5**

**Supplementary Figure S5.** Isoform switch in FHL1 during the D1-to-D21 developmental transition. The upper panel shows transcript structure, predicted coding status, protein domains, and subcellular localization for the four detected FHL1 isoforms. The lower panels show total gene expression, isoform-level expression, and isoform usage, expressed as isoform fraction, at D1 and D21. FHL1 expression was significantly downregulated at D21, while isoform usage shifted toward ENSGALT00010035909.1 as the predominant D21-associated isoform. All detected isoforms were predicted to be coding transcripts encoding cytoplasmic proteins. Asterisks indicate significant differences between developmental stages.

**Supplementary Figure S6**

**Supplementary Figure S6.** Isoform switch in MYH1B during the D21-to-D42 developmental transition. The upper panel shows transcript structure, predicted coding status, protein domains, and subcellular localization for the three detected MYH1B isoforms. The lower panels show total gene expression, isoform-level expression, and isoform usage, expressed as isoform fraction, at D21 and D42. MYH1B expression was significantly downregulated at D42, while isoform usage shifted from ENSGALT00010071401.1 toward ENSGALT00010071405.1 as the predominant D42-associated isoform. All detected isoforms were predicted to be coding transcripts encoding proteins localized to the cytoplasm and/or membrane. Asterisks indicate significant differences between developmental stages.

**Supplementary Figure S7**

**Supplementary Figure S7.** Isoform switch in SMYD1 during the D21-to-D42 developmental transition. The upper panel shows the transcript structure of the two detected SMYD1 isoforms, including predicted coding status, protein domains, and subcellular localization. The lower panels show total gene expression, isoform-level expression, and isoform usage, expressed as isoform fraction, at D21 and D42. SMYD1 expression was significantly downregulated from D21 to D42, while isoform usage shifted from ENSGALT00010038792.1 toward ENSGALT00010038795.1. Both isoforms were predicted to be coding transcripts encoding proteins localized to the cytoplasm and nucleus. Asterisks indicate significant differences between developmental stages.
